# Some considerations in the design and interpretation of antimalarial drug trials in uncomplicated falciparum malaria

**DOI:** 10.1186/1475-2875-5-127

**Published:** 2006-12-22

**Authors:** Kasia Stepniewska, Nicholas J White

**Affiliations:** 1 Faculty of Tropical Medicine, Mahidol University, 420/6 Rajvithi Rd., Bangkok 10400, Thailand; 2 Centre for Tropical Medicine and Vaccinology, Churchill Hospital, Oxford OX3 7LJ, UK

## Abstract

**Background:**

Treatments for uncomplicated falciparum malaria should have high cure rates. The World Health Organization has recently set a target cure rate of 95% assessed at 28 days. The use of more effective drugs, with longer periods of patient follow-up, and parasite genotyping to distinguish recrudescence from reinfection raise issues related to the design and interpretation of antimalarial treatment trials in uncomplicated falciparum malaria which are discussed here.

**Methods:**

The importance of adequate follow-up is presented and the advantages and disadvantages of non-inferiority trials are discussed. The different methods of interpreting trial results are described, and the difficulties created by loss to follow-up and missing or indeterminate genotyping results are reviewed.

**Conclusion:**

To characterize cure rates adequately assessment of antimalarial drug efficacy in uncomplicated malaria requires a minimum of 28 days and as much as 63 days follow-up after starting treatment. The longer the duration of follow-up in community-based assessments, the greater is the risk that this will be incomplete, and in endemic areas, the greater is the probability of reinfection. Recrudescence can be distinguished from reinfection using PCR genotyping but there are commonly missing or indeterminate results. There is no consensus on how these data should be analysed, and so a variety of approaches have been employed. It is argued that the correct approach to analysing antimalarial drug efficacy assessments is survival analysis, and patients with missing or indeterminate PCR results should either be censored from the analysis, or if there are sufficient data, results should be adjusted based on the identified ratio of new infections to recrudescences at the time of recurrent parasitaemia. Where the estimated cure rates with currently recommended treatments exceed 95%, individual comparisons with new regimens should generally be designed as non-inferiority trials with sample sizes sufficient to determine adequate precision of cure rate estimates (such that the lower 95% confidence interval bound exceeds 90%).

## Background

For a patient ill with malaria, rapid resolution of illness without complications from the disease or its treatment is the first priority. Preventing return of the illness is a second priority. In a high transmission setting reinfection is inevitable, so the longer that subsequent illness can be delayed, the better. Those who deploy antimalarial drugs have similar objectives, but need to know more. In particular they need to know the efficacy of the individual treatment against the parasites which caused the infection. The key measure is the cure rate. The cure rate is defined as the proportion of treated patients whose symptoms resolve, parasitaemia becomes undetectable and in whom there are no recrudescences of infection with the genotypes which caused the original illness. This review discusses how the cure rate should be measured and reported.

The treatment of malaria is changing for the better, but this has brought new challenges in the design and interpretation of efficacy assessments. In the past few years, it has become accepted that antimalarial treatments must have high cure rates, which ideally should exceed 90% [1]. The corollary that malaria treatment recommendations should change if cure rates are below 90% requires further definition, but this is a considerable advance on the previous era when much lower rates were considered acceptable, and there was often no reliable information on the cure rates with chloroquine or sulfadoxine-pyrimethamine (by far the most widely used antimalarial drugs). In this earlier context of uncertainty it was reasonable to plan a randomized comparison to test if there was a difference between the regimens being tested (a "superiority" trial). But now better antimalarial drugs exist, and much more information is available about them [2], and thus there is greater a-priori certainty of high cure rates with currently recommended treatments. So as cure rates with current treatments approach 100%, differences between treatment regimens are progressively harder to detect. The conventional "superiority" trial cannot show that a new drug is better. Alternative test strategies are required for evaluating new treatments. Well-conducted, randomized comparative trials are still preferable to single-arm, observational studies as they confirm or refute a-priori estimates of efficacy, reduce investigator biases, and account for systematic errors. Equivalence trials, in which an attempt is made to prove that two (or more) treatments are the same, are unnecessary. The preferable alternative is a "non-inferiority trial", which tests the hypothesis that the new treatment is not significantly worse than the current treatment. It is up to the investigator or current opinion to define the bounds of "significantly worse". But this approach requires different sample size calculations and has certain limitations which will be discussed.

The efficacy of antimalarial drug treatment, in uncomplicated falciparum malaria is assessed by following patients after observed treatment for sufficient time to "capture" all or most of the treatment failures (failures to cure the infection leading to recrudescences) that could occur. As recrudescences result from persistent erythrocytic infection, these recurrent infections re-emerge within a defined time period following treatment [3,4]. This period is dependent mainly on the susceptibility of the infection and the elimination kinetics of the antimalarial treatment. Following treatment with rapidly eliminated drugs most recrudescences occur within four weeks, but following treatment with slowly eliminated antimalarial drugs the recrudescences may be delayed, and so longer follow-up is needed to capture them, otherwise failure rates will be underestimated [5]. Recrudescences more than nine weeks after any treatment are unusual. For this reason, if a rapidly eliminated treatment is compared with a slowly eliminated treatment, and follow-up is only 28 days, the results tend to be biased in favour of the slowly eliminated treatment. Recent studies have characterized the relationship between time to recrudescence and the pharmacokinetic properties of the antimalarial drug treatment, and have provided evidence-based recommendations for the duration of follow-up; 63 days follow-up is recommended for slowly eliminated drugs (t_1/2 _> 1 week e.g. mefloquine, piperaquine) and a 28-day follow-up is the minimum for rapidly eliminated antimalarial drugs [6]. For drugs with intermediate elimination half-lives (t_1/2 _is 1 day to 1 week) 42 days follow-up captures most of the recrudescences. WHO now recommends a minimum of 28 days follow-up in antimalarial drug in-vivo studies. [7]. As increasingly higher cure rates are demanded of new antimalarial treatments, true cure rate must be defined or estimated with better precision. This has important implications for the design of antimalarial drug trials.

Conducting large trials with extended patient follow-up is logistically demanding. In endemic areas it is not possible clinically to distinguish a recrudescence from a newly acquired infection (or, in the case of *Plasmodium vivax *and *Plasmodium ovale *infections, a relapse). The use of PCR genotyping of *Plasmodium falciparum *has considerably improved the ability to conduct community-based clinical trials of antimalarial drugs in endemic areas [8,9]. Unfortunately assessment of treatment responses in *P. vivax *malaria is more difficult as recurrence of the infection may result from recrudescence, reinfection or relapse. The relapses derive from persistent hypnozoites in the liver and are commonly with different genotypes to that identified in the acute infection [10,11]. Assessment in vivax malaria will be subject of a separate review. In *P. falciparum *studies blood samples are taken, usually on filter paper, and the genotypes are compared in blood samples from the acute infection and any infection which recurs during the period of follow-up. *P. falciparum *genotyping is usually based on comparison of variable blocks within the polymorphic genes MSP1, MSP2, and also often GLURP, or by use of microsatellite typing. Sometimes blood samples from the recurrent infection or the acute infection are not available, or go missing, or there are technical reasons while the PCR comparison cannot take place. In higher transmission settings a genotype often cannot be ascribed confidently in multiple infections (i.e. there is patent infection with several different genotypes). How should these patients then be evaluated? There is no clear consensus – but calling all missing or indeterminate results treatment failures is clearly illogical. Ideally in a study, all participants in the trial should complete the study, follow the protocol, and provide data on all the outcomes of interest at all time-points. In reality, most trials have missing data. Data can be missing either because critical information, such as PCR genotyping, is missing or uninterpretable [12], or because some of the participants drop out (i.e. fail to attend for the follow-up appointments) before the end of the trial. This risk increases with longer follow-up periods, long after patients have finished treatment and benefited from it. Patients with missing data are often considered conservatively as therapeutic failures (an "intention to treat" or "ITT" approach), but with highly efficacious treatments as failure rates approach zero, missing data may comprise the majority of "therapeutic failures" thereby distorting considerably the assessment of efficacy. In this review some suggestions (with formulae and worked examples) are presented for the design of antimalarial drug trials and the analysis of incomplete data.

### Failure data are "survival" data

In antimalarial drug trials there are two or more groups of patients followed for a prespecified length of time after different antimalarial treatments. The cure rates, which means the proportions of patients who reach the end of this follow-up period without recrudescence of the infection are compared. In the past, antimalarial treatment efficacy was usually assessed on a particular day (often day 14 or day 28 after starting treatment) so only patients followed to that day were included in the analysis. This is often referred as a ***"per-protocol" (PP) analysis***. But in most trials there are patients who do not complete the follow-up period, yet these patients do contribute useful information before they leave the trial, and this can and should be used. If such a patient did not fail (i.e. remained aparasitaemic) when last observed, that patient's data are said to be 'censored' at the time they were last followed up. The appropriate analysis for such data is ***survival analysis***. This analytical approach is well established in the assessment of cancer chemotherapy and increasingly in the assessment of anti-infective drugs, as survival analysis deals explicitly with censored values. Patients with different follow-up periods cannot be treated the same way – someone who is followed up for longer has a greater chance of being recorded as treatment failure than another patient followed up for a shorter time. Failure rates should be estimated using the Kaplan-Meier method [13]. This is now endorsed by the recent WHO recommendations for antimalarial resistance monitoring which suggest use of life tables (i.e. survival analysis) in analysing in-vivo studies [7].

### Estimation of failure rates

The proportion of subjects beyond any follow-up time ***t ***who have not developed a recrudescence is estimated by the Kaplan-Meier method as

p = ∏ (r_i_-d_i_)/r_i_

where r_i _is the number of subjects without a recrudescence just before time t_i_, d_i _denotes the number who had a recrudescence at time t_i _and ∏ represents the product of all the estimates at each time point t_i _until time ***t***.

This proportion is equivalent to the efficacy of the treatment and 1-p is equivalent to the failure rate of the treatment.

The following example illustrates how analysis of categorical data at defined end-points leads to errors when patients are lost to follow-up and how this is best dealt with by survival analysis.

#### Example

In a study of an antimalarial drug, follow-up was for 63 days. 100 patients were enrolled, and all of them were evaluated at 28 days, but thereafter only 60 were observed at each appointment until day 63. At day 28, 21 failures were observed (and these patients were therefore not followed further), an additional four failures were recorded at day 63, and 19 patients were lost to follow-up between days 28 and 63.

Estimation of the failure rate at day 28 is easy and equals 21/100 = **0.21**.

What is the day 63 failure rate?

If all patients had been followed up until day 63 then the failure rate would have been 0.25 (25/100), but it was not the case.

In total 25 failures were observed but patients were followed up for different periods of time. An analysis done only on patients who completed the follow-up would have a denominator of only 60 as the early failures (N = 21) and the patients lost to follow-up (N = 19) are not included. It is obviously wrong to ignore these early failures, but if they are included in the analysis it assumes that patients excluded (i.e. the 19 lost to follow-up) had the same probability of failing during the entire follow-up period as those included, whereas those lost to follow-up did not fail in the period between 0 and 28 days (when 21 of the 25 observed failures did occur). The failure rate would be estimated as:

25/(60 + 21) = 25/81 = 0.31     (A)

The correct analysis is based on the Kaplan-Meier approach which incorporates these temporal changes in the probability of failing treatment.

In the example, the failure rate at 28 days is 21/100, so the probability of not failing during 28 days is 1-(21/100) or **0.79**.

The 60 patients who did not fail until day 28 were followed up until day 63, and for them the probability of failing between day 28 and day 63 is 4/60 or 0.0667. Thus, the probability of not failing between days 28 and 63 is 1-0.0667 or **0.933**.

Of course patients can only not fail in the second interval (28–63 days) if they did not fail in the first interval (0–28 days). Therefore, the probability of not failing during 63 days is equal to the product of the probability of not failing during 0 to 28 days and the probability of not failing between 29 and 63 days. This product is the treatment success rate and the failure rate is one minus the success rate.

So the failure rate is estimated as :

1-(1–21/100)*(1–4/60) = 0.27     (B)

This estimate is significantly lower than the per-protocol estimate. Splitting follow-up into intervals and calculating failure rates for these intervals makes sense biologically. Recrudescences do not have the same probability of occurring across the entire period of follow-up.

**Figure 1:**
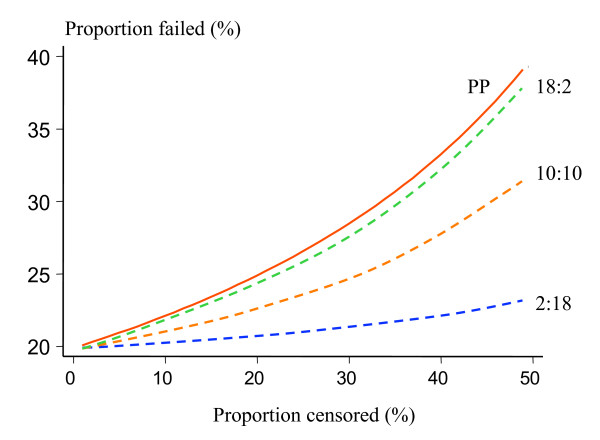
Comparison of the effects of trial drop-outs on estimated failure rates in 100 patients followed for 63 days after a treatment with a true 20% failure rate. The "per protocol" method (PP), of estimating cure rates in which the denominator at each time point is the combined number of patients who were followed until that point and continued to be aparasitaemic and those who had a recrudescence observed, is shown as a red solid line and compared with the Kaplan-Meier survival method. The drop-outs all occurred after 28 days of follow-up. Three different scenarios are presented with differing proportions of failures (20 in total) presenting before and after 28 days: blue dash line – 2 failures before and 18 after day 28; orange dash line – 10 failures before and 10 failures after day 28; green dash line – 18 failures before and 2 failures after day 28.

Figure 1 compares the per protocol estimate A (PP) of the failure rate and the Kaplan-Meier estimate B (KM) for different numbers of patients dropping out from trial follow-up after day 28 and different distributions of observed failures. It is assumed that 100 patients were followed up until day 28, and 20 failures were observed in total. The x-axis represents the percentage of patients who dropped out after day 28. Three scenarios are considered: (a) the observed failures were equally spaced in time, 10 before and 10 after day 28; (b) more failures (n = 18) occurred before day 28; (c) more failures (n = 18) occurred after day 28.

For all three distributions of observed failures, the PP rate estimate is the same, but the KM estimate varies. The discrepancies between the two methods are greatest when most of the observed failures occurred before patients were lost to follow-up. Discrepancies also increase dramatically with the proportion of patients lost to follow-up. Guthmann *et al *have recently reported a comparison of per protocol and survival analysis [14] in 13 paediatric studies conducted in sub-Saharan Africa. Only 6% of patients data were lost with the KM survival analysis, compared with 25% with the PP analysis. In high transmission settings, or where mixed infections with *P. vivax *are common, a very high proportion of patients may have a recurrent infection within the follow-up period and be lost to a PP analysis [12].

### Assessment of confidence intervals

Statistical software used for the calculation of Kaplan-Meier estimates will usually also provide confidence intervals; these will be Greenwood's confidence intervals or confidence intervals based on the asymptotic variance of -log-log transformation of the survival function [15]. However it should be kept in mind that Greenwood's method underestimates the variance of the Kaplan-Meier estimate. Alternatively the confidence intervals can be calculated using the effective sample size suggested by Peto [13]. 'Effective' sample size at time t_i _will be equal to the total number of patients when there are no censored observations, that is when all patients were followed until the recurrence or time t_i_. When there are censored observations then the effective sample size at time t_i _is

N' = (r_i_-d_i_)/p;

where r_i _is the number of subjects without a recrudescence just before time t_i_,

d_i _denotes the number who had a recrudescence at time t_i _and p is the Kaplan-Meier estimate of a proportion of patients surviving without a recrudescence beyond point t_i_.

If the failure rate is very small or zero, standard confidence intervals for proportions based on a normal approximation are not appropriate [16,17] and should not be used. Wilson's method [18] is recommended for small proportions while the exact binomial method can be used for zero values:

0 to 1-(á/2)^1/N'^;

where á is a significance level and N' is the effective sample size. An Excel spreadsheet for calculating the confidence intervals for proportions and their differences is freely available on the web [19].

### Comparing the treatment groups

The simplest way of comparing and presenting the failure rates and failure times between the treatment groups is to plot the Kaplan-Meier survival estimates on the same axes. To distinguish between chance variation in failure rate estimates in the two groups and a real difference, a hypothesis test is required. For survival data, two tests are commonly used: the log-rank test and the Wilcoxon test. Both test the null hypothesis that there are no real differences between the two groups, so small values of the test statistics will correspond to the acceptance of the null hypothesis. Both are based on the sum of the differences between the observed and expected number of failures in each group over all time points, but in the Wilcoxon test this sum is weighted by the total number of individuals at risk at each time. The log-rank test is preferable when the Kaplan-Meier plots for the two groups do not cross, as this reflects a continuous proportional difference in failure rates between the two groups. If the survival curves do cross, the Wilcoxon test should be used.

It is always useful to express the difference between the two treatment groups in a summary measure. As the risk of failure changes over time and the rates of change are usually different in the two treatment groups, only measures evaluated at a prespecified timepoint are recommended. These could be the absolute risk reduction or the risk ratio (or relative risk). Hazard ratios [20], which are commonly used in cancer treatment studies are usually not relevant to malaria drug studies as they are assumed to be constant across time.

The absolute risk reduction is the difference between failure rates in the two treatment groups. The risk ratio (relative risk) is calculated as the ratio of those failure rates. In antimalarial trials, because of losses to follow-up, Kaplan Meier estimates of failure rates should always be used. The absolute risk reduction assesses the clinical importance of the treatment difference while the relative risk has the intuitive appeal as it measures the magnitude of the difference.

However, there are pitfalls in using the relative risks – for uncommon events, large relative risks will result from differences of only a few failures between the treatments, for example 2% difference in failure rate between 2 treatments of efficacy rates of 97% and 99% gives relative risk of 3.

### Proving non-inferiority in antimalarial drug comparisons

**Figure 2:**
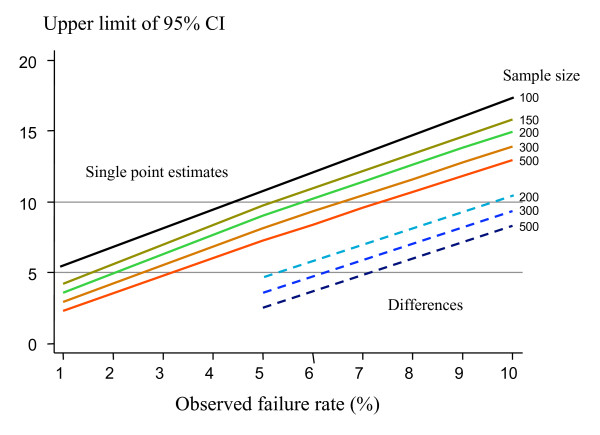
The upper limit of two sided 95% confidence intervals for single point estimates of failure rate for different sample sizes, estimated using Wilson's method, are shown as solid lines (upper left). The upper limit of two sided 95% confidence intervals for differences between the observed failure rate and a standard treatment with a failure rate of 5% for different sample sizes, estimated using Newcombe's method, are shown as dashed lines (lower right).

In the past antimalarial drug trials have been powered to detect differences between drugs – usually with 95% confidence and 80% power. This is increasingly difficult at cure rates over 90% because of the exponential increase in the sample size required (Figure 2). The higher the standard treatment's cure rate, the more difficult it is to demonstrate conclusively a small difference in favour of a new treatment. Characterizing a small difference in cure rates (e.g. between 96% and 99%) is potentially important (after all, this represents a four-fold difference in failure rates) but it may not be logistically feasible. An alternative approach is the non-inferiority trial. There is no clear point at which superiority trials must give way to non-inferiority trials. This depends on how the results of the trial are to be used.

Non-inferiority trials aim to show that an experimental treatment is not worse than the active control (i.e. current treatment) by more than a specified amount – the equivalence margin (often denoted δ). The null hypothesis being tested is that there ***is ***a difference between the two groups (i.e. it is the opposite to that in conventional superiority trials) and it is greater than the δ. Rejection of the null hypothesis indicates that there is no difference between the groups. The choice of an appropriate value for δ is a compromise based on current knowledge, clinical judgement, likely policy implications and the practicalities of conducting large trials. Values of 10% have been widely used in assessing antimicrobial agents, but are too wide for current requirements for antimalarial drug efficacy. Smaller equivalence margins require that studies have larger sample sizes that have been usual in the past [2]. Each comparison should be considered individually [21,5]. There is a burgeoning statistical literature on the limitations of non-inferiority trials, much of it recent [22-24]. The main limitation from a statistical perspective is that confounders introduced in a poorly conducted trial which affect both groups, and are unrelated to differences in the efficacy (or toxicity) of the trial regimens, can obscure significant differences. In a superiority trial this might lead to a failure to disprove the null hypothesis – i.e. failure to show difference – but in a non-inferiority trial the direction is opposite; a false rejection of the null hypothesis and conclusion of non-inferiority [25]. This emphasizes the importance in antimalarial drug trials of avoiding errors in drug allocation and administration, poor adherence, errors in end-point ascertainment (for antimalarial efficacy this refers particularly to identification of recrudescence), and loss to follow-up. These considerations are particularly relevant to the choice of analytical approach. The intention to treat (ITT) approach, which is a robust, albeit conservative, method of assessing superiority, is particularly vulnerable. It should not be used as the primary endpoint for the assessment of antimalarial drug efficacy when cure rates are high.

Blinding is often used to avoid bias in comparative trials although it is often difficult in antimalarial drug assessments because of differences in treatment regimens and the difficulties in masking the taste of the drugs. Compared with superiority trials, blinding does not protect against bias as well in non-inferiority trials because a biased investigator wishing to show non-inferiority can simply give all patients similar results! Analysis of non-inferiority trials requires a calculation of the difference between the failures rates in the treatment groups and a calculation of the confidence interval around this difference using appropriate methods [26] and 'effective' sample sizes. An example is provided in the Appendix 1.

### How should the results be reported?

Intention-to-treat (ITT) analysis intends to include all patients randomized into the trial irrespective of what happened to them subsequently [27,28]. This is straightforward if patients' outcomes have been evaluated and the violation of the protocol was with respect to the received treatment. But if their outcome is unknown there is no clear consensus if patients should be still included in the analysis [29], especially if their inclusion is only possible after some imputation of the outcome is performed.

In the per protocol analysis (PP), on the other hand, drug trial results are often analysed simply in categorical tables comparing proportions of patients deemed to have been cured at the predefined trial time-point(s). As explained previously this ignores the contribution of information provided by patients whose follow-up was incomplete.

**Figure 3:**
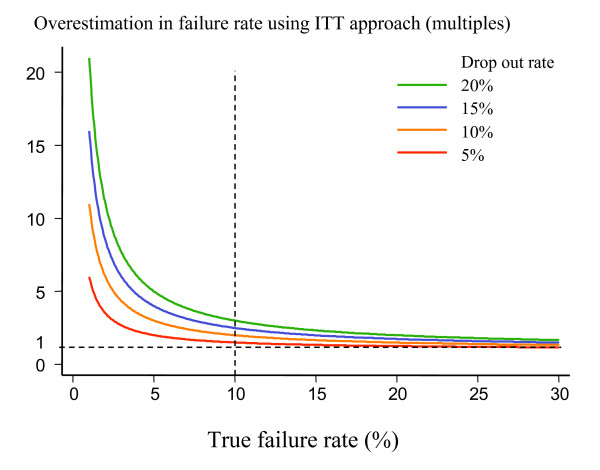
Degree of overestimation of the true failure rate provided by an ITT analysis where all patients failing to complete the trial satisfactorily are considered as "treatment failures". Overestimation is calculated as a ratio of estimated failure rate using the ITT approach to the true failure rate. When true cure rates are low (i.e. less than 10% – vertical dotted line) the overestimation is considerable.

The intention-to-treat analysis (ITT) analysis is widely used to generate the most conservative estimate of the efficacy of an antimalarial drug. In an ITT analysis all patients who do not complete the follow-up period successfully may be considered therapeutic failures. The results then provide a combined assessment of the trial conduct and the drug efficacies, with efficacy and follow-up failures both having equal weight in the analysis. In antimalarial drug trials with long follow-up drop-outs increase with time. Thus, even if a drug has 100% true efficacy, an ITT analysis will show progressively worse results as the duration of follow-up is extended. It is simply measuring loss to follow-up and indeterminate or missing genotyping results. Slowly eliminated drugs which require longer follow-up will always fare worse. The better the drug is (ie. the higher the true cure rate), the greater is the proportional difference between true cure rate and ITT estimate. This is illustrated in Figure 3. The ITT analysis should still be done and reported to illustrate a "worst-case scenario" but it should not be the primary endpoint in large community-based trials of antimalarial drug efficacy where long follow-up is required and drop-outs likely.

### Missing data problems

#### a) How should we deal with missing appointments?

Some patients miss a follow-up appointment and then attend subsequently. These patients should not be censored from efficacy assessments if they have remained well, as it is unlikely that a recrudescent malaria infection would have occurred during the missed appointment period which then rapidly and symptomlessly self-cured. If the patient presents after the missing appointment with a recrudescence then the history can be used to estimate the onset of recrudescence. Continuous data (e.g. gametocyte-time curves) present more of a problem, and these need to be assessed on an individual basis. There may be sufficient data to justify interpolation, or omission may be appropriate.

#### b) Interpretation of indeterminate PCR genotyping results

The use of genotyping to distinguish reinfection from recrudescence is based on the relative probabilities of finding identical polymorphic malaria parasite alleles by chance from the parasite population. It is necessary to define these probabilities for each individual allele for the parasite population (and thus patient population) under study. For multiple alleles their individual distributions must be unlinked. If there are multiple genotypes present then qualitative assessments (without extensive sequencing) cannot ascribe an individual genotype, and although probabilities can still be ascribed to recurrent infections, statistical power is reduced. If a patient has a recurrent infection in an antimalarial drug trial conducted in an endemic area, but a paired sample is either unavailable or, for technical reasons (no amplification, multiple bands precluding a definitive result, etc.), a comparison of genotypes cannot be made then a recrudescence (treatment failure) cannot be distinguished from a newly acquired infection. The interpretation of genotyping results in high transmission settings, and the possibility that minority (undetected) genotype populations may cause subsequent recrudescence is a subject of considerable interest and debate. This is important subject and advances both from a biological and statistical perspective can be expected in the near future. But for the purpose of this discussion it is simply accepted that a potentially large number of patients may have indeterminate results, and that there is uncertainty as to how these patients should be analysed.

To present the different analytical approaches, first it is necessary to describe the following proportions:

At start of each interval (t_i_, t_i+1_, etc) there are n_i_, n_i+1_, etc. patients at risk of having a recurrence of parasitaemia.

If during the interval from t_i _to t_i+1 _there were

r_i _patients with recrudescent infections,

a_i _patients with newly acquired patent infections,

c_i _patients without patent infections (i.e. aparasitaemic) and

d_i _patients who were lost to follow-up

then

n_i _= r_i _+ a_i _+ c_i _+ d_i........................................................................................................................................... _(1)

Then n_i+1 _= n_i _- (r_i_+ a_i _+d_i_) as those patients with true recrudescences previously are no longer "at risk" (i.e. they cannot have another recrudescence), and both dropouts and those with new genotype infections are not followed up further (the latter group having been treated).

The observed recurrences (or_i_) for whom genotyping results are available will be either recrudescences, or new infections, but for some of them the PCR results will be indeterminate and so their status will not be known. If indeterminate PCR genotyping results (ind) are unrelated to treatment failure rates, and are not more or less likely in recrudescences than in newly-acquired infections, then they will occur at a constant rate (i.e. a constant proportion (f) of the total recurrences with time):

ind_i _= f·(r_i _+ a_i_)).....................................................................(2)

and subsequently ∑ ind_i _= f·∑ (r_i _+ a_i_)...........................................(3)

In this notation, there are (1-f) r_i _confirmed recrudescences and (1-f) a_i _confirmed new infections and so as or_i _= r_i _+ a_i_

then or_i _= (1-f) (r_i _+ a_i_) + ind_i _.......................................................(4).

At each time interval, numbers r_i _and ind_i _are small so estimates of f may be inaccurate, and it may be better estimated from the equation (3) using the total number of indeterminate results and the total number of observed recrudescences.

**Figure 4:**
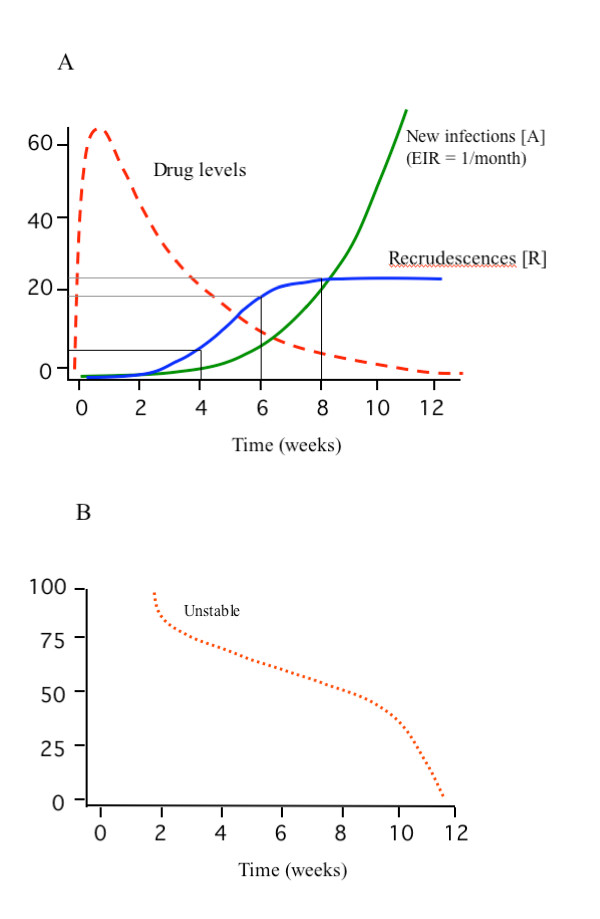
a and b. Simulated example of a clinical trial evaluation of a slowly eliminated antimalarial drug (e.g. mefloquine) with a 23% failure rate evaluated in an area with high malaria transmission (EIR 12/year) (upper panel). The apparent failure rate based on genotyping at 4 weeks is 3% and at 6 weeks is 15%. All patients are eventually reinfected once the drug has been eliminated and the prophylactic effect exhausted. There is a non-linear relationship between recrudescences and reinfections. Figure 4b shows the proportion of recurrent infections that are recrudescences.

The ratio of recrudescences to new infections will depend on the failure rate (which for new treatments should be less than 10%), the malaria transmission intensity, and time since the start of the treatment. In high-transmission settings, eventually all patients will be reinfected. This and the non-linear relationship between recrudescences and reinfections (reflecting the relationship between a discrete and a cumulative distribution) are illustrated in an example which follows and in Figures 4a and 4b. But this changing proportion of new infections and real recrudescences does not affect the rate f. If f is only related to the PCR technique and not malaria we would still have f·(r_i _+ a_i_) indeterminate results. So how should indeterminate results be treated in the analysis of efficacy?

##### (i) Treating indeterminate results as failures (ITT or the worst-case scenario analysis)

In this approach all indeterminate PCR results are treated as recrudescences (i.e. treatment failures).

i.e. the failure rate for interval (t_i _to t_i+1_) is:

F_A _= ((1-f)r_i _+ ind_i_)/(n_i_)

Unless there are no reinfections this method overestimates the failure rate. This overestimation (proportionally) is not so profound at high failure rates or at low levels of malaria transmission, but, unless rates of indeterminate results are low, it creates serious discrepancies at low failure rates in areas of medium to high transmission. At its most extreme, if all patients are followed until they acquire their next infection, then for a drug with 100% efficacy (no true failures) the ITT failure rate equals the rate of indeterminate PCR results. As this could well exceed 10% then even a completely (100%) efficacious antimalarial drug cannot achieve the level of >90% cure rate currently recommended by the World Health Organisation in the trial [1]. The effects of treating indeterminate results as failures on the cure rate estimate are shown in Tables 1 and 2. With increasing numbers of indeterminate results the intention to treat analysis progressively overestimates the true failure rate. The errors are proportionately greatest at low failure rates. The ITT approach to handling missing or inderminate PCR results is therefore incorrect.

**Table 1:** Illustrating how the "Intention to treat" approach ascribing indeterminate treatment outcomes as failures overestimates the true failure rate. High failure rate:

Follow-up	A (%)	R = F(%)	F_ITT _(%)	**Overestimation of failure rate (%)**
6 weeks	6	15	15.3	2%
8 weeks	20	25	26	4%
10 weeks	45	25	27.25	9%
12 weeks	68	25	28.4	14%
20 weeks	75	25	28.75	15%

Assume that the entomological inoculation rate is 1/month (Figure 4), the true failure rate (F) is 25%, and 5% of PCR pairs are indeterminate. The patients are censored when a recurrent infection occurs.

Then at 4 weeks follow-up in the trial, the recurrence rate = 3.5% (2.5% true recrudescence, 1% true recurrence)

True failure rate = 2.5%, ITT analysis failure rate = 2.55%, overestimation 1.275%. F_ITT _(%) = A + 0.05 R

A – cumulative probability of developing a patent new infection

R – cumulative probability of developing a patent recrudescence

**Table 2:** Illustrating how the "Intention to treat" approach ascribing indeterminate treatment outcomes as failures overestimates the true failure rate. Low failure rate

Follow-up	A (%)	R = F(%)	F_ITT _(%)	**Overestimation of failure rate (%)**
6 weeks	6	3	3.3	10%
8 weeks	20	5	6	20%
10 weeks	45	5	7.25	45%
12 weeks	68	5	8.4	68%
20 weeks	75	5	8.75	75%

If the failure rate is low (as it hopefully should be) the errors using the ITT approach are very large. In this example everything is the same as in the above example but now the true failure rate (F) is 5%,

A – cumulative probability of developing a patent new infection

R – cumulative probability of developing a patent recrudescence

##### (ii) Treating indeterminate results as censored

In this approach no assumptions are made, and the patients are simply censored from the analysis. They can be censored at the time when the recurrent parasitaemia occurred, that is at the end of the interval, at time t_i+1_.

F_B _= (1-f) r_i_/n_i_

Patients with the indeterminate results are treated in exactly the same way as patients who became smear negative after treatment and were then lost to follow-up.

But as the PCR result is not known, and therefore whether it is a recrudescence or reinfection cannot be determined, the standard approach in survival analysis to the patient's data would be to exclude them from the analysis in the interval when the recurrence took place, so they have also to be deducted from the number at risk n_i _at the beginning of the interval.

F_C _= (1-f) r_i_/(n_i_-ind_i_)

This corresponds to censoring them at the end of the previous interval, at time t_i_.

It could be argued that this type of censoring is not non-informative but if the number of patients lost to follow-up is small, very little bias is likely to result from applying methods based on non-informative censoring [30].

##### (iii) Adjustment of the number of failures by the time adjusted rate of true failures derived from the valid PCR genotyping

This approach uses all the available data but relies on there being sufficient data to characterize the temporal changes in the probability of recurrent parasitaemia being a recrudescence (g_i_) where g_i _is the proportion of recurrent infections at time t_i _which are recrudescences. Thus at each time point for a recurrent parasitaemia this probability of recrudescence (g_i_) and a probability of reinfection (1-g_i_) are determined for the study population from the valid PCR-genotyping results. Obviously this requires sufficient data for adequate characterization. This ratio of probabilities is then applied to any indeterminate results.

F_D _= or_i _g_i_/n_i _= (or_i_·(1-f) r_i_/(or_i _- ind_i_))/n_i _= F_B_/(1-f)

where g_i _= (1-f) r_i_/(or_i _- ind_i_) is the proportion of recurrences with confirmed PCR results which are recrudecences at time t_i_.

Estimate F_D _is the most accurate provided we have a good estimate of g.

Estimate F_C _is methodologically correct but its includes patients with indeterminate results only while they did not have recurrent infection. There is currently no consensus on which approach should be taken, or the precise modelling approach to the calculation of g.

### Sample-size considerations

The assessment of efficacy of an antimalarial treatment is based on the observed cure rate and the confidence intervals around the estimate. In a comparison of two treatments we calculate the individual confidence intervals around the cure rates observed, then the difference between the cure rates, and then the confidence interval for the difference. The sample size determines the width of these confidence intervals. Confidence interval calculations based on the normal approximation (for estimation of the standard error) are not appropriate for very small proportions (i.e. very low failure rates). The methods of Wilson [17,18] and Newcombe [26] are preferable in the case of a single proportion and multiple proportions respectively.

Upper limits for 95% confidence intervals using Wilson's method are presented in Figure 2 (solid line), for different sample sizes and for treatment failure rates ranging between 1 and 10%. Sample sizes of more than 150 give an upper 95% confidence interval limit of less than 10% when the observed failure rate is 5%. In the same figure upper 95% confidence interval limits for the difference between the observed cure rate of the new treatment and the 95% cure rate of a standard treatment are presented derived by Newcombe's methods.

**Figure 5:**
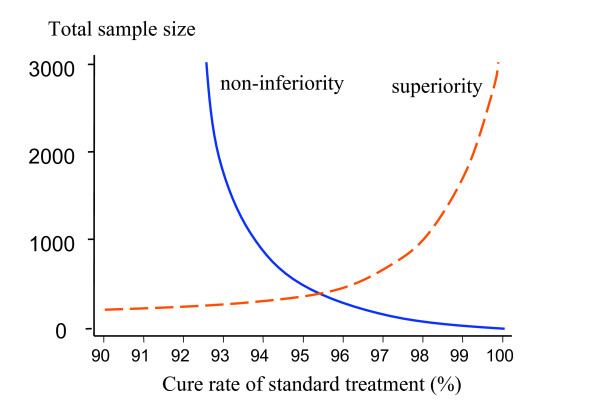
Operating within the confines of cure rates which should exceed 90%, and thus trials which must have the statistical power to exclude, with 95% confidence, that the cure rate of the new antimalarial does exceed this value, the relationship between sample size (y axis) and cure rate of the established treatment (x axis) is illustrated. For superiority trials with 2α= 0.05 and β = 0.2, the dashed line shows the ***lower limit ***of possible sample sizes; i.e. when the new treatment has an estimated efficacy of 100%. For non-inferiority trials the solid line shows the sample size required to ensure that the 95% CI for treatment efficacy of the new treatment exceeds 90%; i.e. the non-inferiority margin δ is (cure rate of the standard treatment-0.9), assuming that the true cure rates are the same. These assume 1:1 randomization.

The above calculations are based on the assumption that there are no losses to follow-up. But if there are losses, the 'effective' sample size of Peto [12], which is defined later in this paper, should be used in sample-size calculations. As mentioned previously – the sample size should be chosen on the basis of the estimated true cure rate. In all calculations in this paper improved methods for confidence intervals are presented (and recommended), as the standard methods based on the asymptotic normal approximation exhibit poor coverage, especially for small proportions. Figure 5 illustrates the relationships between sample size and power to detect superiority, and non-inferiority within the confines of cure rates between 90% and 100%.

### Unequal randomization?

Unequal randomization, for example 2:1 randomization, may also be considered in trials assessing new treatments. Although unequal randomization sacrifices statistical power slightly (or requires larger total sample size) in the comparison, it increases the precision in the estimate of treatment efficacy of the new drug, and it also provides a better adverse-effect characterization for the new treatment. The "control" (i.e. current treatment) trial arm is still important in helping to distinguish "trial" from "drug" problems i.e. it helps to identify systematic trial-related confounders which lead to unusual efficacy or toxicity findings. For example, a trial in which non-inferiority was shown but both treatment arms performed poorly (i.e. less than 90% cure rates) would not warrant rejection of the new treatment if the prior information suggested much better efficacy of the established treatment. There might have been a problem in the conduct of the trial. Thus there is a statistical trade-off between both the characterization of the difference between the two regimens and the precision of the "control" arm estimate, and the characterization of the new treatment effects. But there are also biological and programmatic reasons why it may be important to have a larger sample size for the new antimalarial treatment; as cure rates asymptotically approach 100% the selective force that drives the emergence and spread of resistance weakens, and in low transmission areas, provided coverage is high, the incidence of malaria will fall. Precise characterization of very high cure-rates provides important information to the policy maker which will be taken into account with costs, simplicity of administration, tolerability, adverse effect profile, etc., to influence the difficult decision of whether or not to change treatment recommendations.

**Figure 6:**
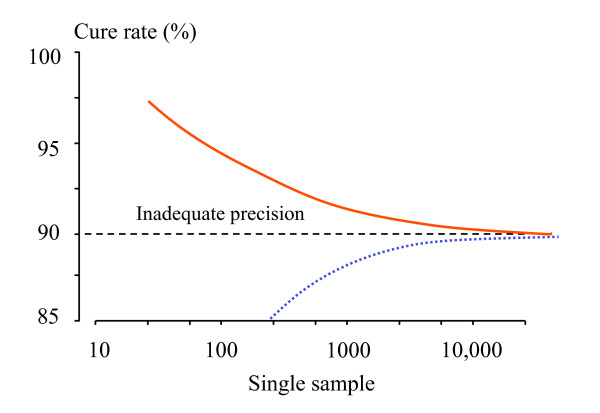
The relationship between sample size (in a single sample) and precision in characterizing the cure rate is shown; the upper solid line shows the boundary for the cure rate below which the lower 95% confidence interval bound for the proportion falls below 90%, and the lower dotted line shows the boundary for the cure rate below which the upper 95% confidence interval bound for the proportion exceeds 90%. Sample sizes to the left of the curves provide inadequate precision if the objective is to be sure the confidence interval for the sample does not cross 90%.

It is evident from Figures 2, 5, and 6 that large sample sizes are required to provide sufficient precision when efficacy is high. Maximum likelihood methods can be used to adjust the balance of unequal samples to optimize the information gained [31].

### Practical realities

If the existing recommended antimalarial treatment used in comparative assessments of new antimalarials is still highly efficacious then sample sizes in conventional superiority trials must be large. For example, if the true cure rate for a new antimalarial treatment exceeds 99%, then the total sample size required to show superiority over the existing treatment, if this has an efficacy of 95%, exceeds 650. This doubles to over 1,300 if the new treatment is 98% efficacious. A 2:1 randomization increases these numbers by <8%. Few single centre antimalarial drug trials enrol more than 650 patients in a study. This seems to be the limit for superiority testing. If a non-inferiority trial is conducted, and the differences are much larger than anticipated, then a significant difference may be demonstrated. If the new compound has a significantly lower cure rate this would argue against its introduction. For the established compound more investigation might be necessary to determine the reasons why it performed less well than expected.

## Discussion

The outlook for antimalarial chemotherapy has improved in recent years with the introduction of several new highly effective drug combinations, and elevation in the cure rates now required of these new treatments. A cure rate of at least 90% in uncomplicated malaria assessed at 28 days is recommended by the World Health Organization [1]. As a result antimalarial drug evaluations will increasingly be performed with highly effective current treatments as comparators. This makes the sample-size requirements for conventional superiority trials logistically difficult or simply impossible (Figure 5). Furthermore, demonstration of superiority by a small margin (by definition only a few percentage points), although potentially important in terms of resistance prevention, may be offset in operational terms by unrelated factors such as cost, simplicity of dosing, or adverse effects profile. It is worth noting that cure rate targets in drug development (i.e. phase 3) can be lower than in operational use, as there may still be improvements possible (particularly in dosing) which would increase the cure rate. But once the drug formulation and dosing have been optimized cure rates should ideally exceed 95%, and showing superiority within the 95 to 100% cure rate range is unlikely to be logistically feasible. Non-inferiority trials are alternatives to superiority trials which provide valuable information, but require a different approach to sample-size calculations, and are very vulnerable to confounders introduced by poor conduct of the study. As antimalarial drug trials employ longer follow-up periods to characterize better antimalarial drug efficacy, the problems with missing or indeterminate data will increase. Incorrect analysis of these data (particularly calling all missing or indeterminate results "failures") may lead to significant overestimates of treatment failure rates, and could even lead to inappropriate discontinuance of an effective treatment. Standardizing analytical approaches is as important as standardizing clinical trial methodology. The intention to treat (ITT) approach to analysis of efficacy, which treats patients with incomplete follow-up and other protocol deviations as treatment failures, is widely recommended as it provides the most conservative estimates of efficacy, and thereby reduces the possibility that bias may favour one of the treatments. The ITT analysis should be reported in comparative trials of antimalarial drugs, as it gives an unbiased assessment of differences and provides a comparison of effectiveness and toxicity leading to trial discontinuation, but it should not be the primary end-point used as a basis for sample-size calculations, or for reporting efficacy assessments in uncomplicated malaria. This is because practitioners and policy makers need to know true failure rates, and at the low failure rates now required of new antimalarial drugs, the ITT analysis considerably overestimates the true failure rate if there are patients who do not complete the study (Figure 3). Effectiveness is certainly the most important parameter in assessing antimalarial drugs, but to interpret ineffectiveness it is necessary to know efficacy. Omitting patients with incomplete follow-up from the denominator in failure rate estimates also leads to significant overestimates, particularly if the majority of failures occur early in a trial. In non-inferiority trials where there are protocol deviations and incomplete follow-up the ITT approach may lead to a false conclusion of non-inferiority. To estimate antimalarial drug efficacy the survival analysis approach provides the best comparative estimates of therapeutic efficacy. It is well established in non-infectious diseases, and should be used more in the assessment of all infectious disease treatments where the assessment of treatment failure rates requires long follow-up.

There has been a considerable improvement in the quality and quantity of antimalarial drug trials reported in recent years. The introduction of PCR genotyping [8] has allowed large community based studies to be conducted in patients of all ages. In the low-transmission settings where it was first used there have been relatively few problems with interpretation. But at high levels of transmission intensity multiple genotypes are usual and without quantitative methods, it may be difficult or impossible to ascribe genotypes accurately [32]. Better genotyping methods are being developed but, for the present, most investigators rely on simple PCR with analysis of bands on gel electrophoresis. This uncertainty, which makes confident distinction of a recrudescence and reinfection difficult or impossible, has led some investigators to suggest that for trials in high transmission areas, genotyping should be abandoned – arguing that reinfection and recrudescence are of equal importance. This remains to be proved. Such an approach would require a fundamental change in the perspective on treatment (placing much greater weight on post-treatment prophylactic effect). In an artemisinin based combination treatment resistance to the slowly eliminated partner drug will reduce the average duration of the post-treatment prophylactic effect and increase the probability of recrudescence. The relationship between these two related effects depends on several independent variables, and has not been well characterized for any antimalarial drug. But while the jury remains out on the relative importance of curative efficacy and the post-treatment prophylactic effect, and more evidence is accrued, treatment trials generally include genotyping – and therefore will provide indeterminate or missing PCR results. The ITT approach to analysis where all missing or indeterminate PCR results are treated as failures, although often undertaken, is obviously wrong. The longer the follow-up, the greater are the chances of reinfection, and the more PCR genotyping will be required. This will produce correspondingly more indeterminate or lost results and greater overestimation of the true failure rate – all of which is independent of drug efficacy. There are two possible approaches to resolve the problems this creates; either these patients should be omitted from the calculations (this preserves the correct difference between the groups but gives imprecise individual drug efficacy estimates), or preferably if there are sufficient data then a survival analysis approach should be taken, and the time adjusted probabilities of recrudescence versus reinfection should be calculated from the valid genotyping pairs. Thus a data driven probability can be ascribed at any time point to a recurrent infection being a recrudescence. Both approaches are compromised if patients who "drop out" from trials or have missing or indeterminate PCR values are unrepresentative of the remaining patients (i.e. they are more or less likely to fail treatment than the rest). This will need to be evaluated locally. The greater the number of missing values the less confidence there will be in the results of the trial. Standardization of methodologies and consensus recommendations on analytical approaches would help the malaria researchers and control programmes.

With the increasing efficacy of new treatments and requirement to aim for cure rates of > 90% and preferably ≥ 95%, comparative trials should generally be designed as non-inferiority trials in which the null hypothesis is that there is a difference between the two groups when existing treatment efficacy still exceeds 95%. These trials should be powered to give a predefined precision for estimates of cure rates. For consideration as a policy option the point estimate of the cure rate of the new drug treatment, and the currently recommended treatment should both exceed 90% as this is the threshold currently recommended by WHO. Large trials are required to provide adequate precision of these estimates.

## Conclusion

Antimalarial drug comparisons must be large enough to provide precise cure-rate estimates, and follow-up must be long enough to capture the majority of recrudescences. Non-inferiority trials may be necessary when standard treatment efficacy is high (cure rates over 90%), but these have weaknesses which may not be familiar to investigators used to superiority trials. The primary efficacy endpoint should be derived from survival analysis. Intention to treat and per protocol point-analyses should be reported also as secondary results. The interpretation of current PCR gel-electrophoresis derived genotyping results in high transmission settings is difficult. Indeterminate or missing results should not be classified as treatment failures, but should also analysed using a survival approach. Consensus recommendations on the interpretation and analysis of antimalarial drug trials would be of great benefit.

## Abbreviations

α – significance level

δ – equivalence margin

∏ – multiplication symbol

∑ – summation symbol

A – cumulative probability of developing a patent new infection

a_i _– patients with newly acquired patent infections at time ti

c_i _– patients without patent infections at time ti

CI – confidence interval

d_i _– patients were lost to follow-up at time ti

F – true failure rate

f – proportion of the total recurrences which have an indeterminant PCR result (true value is constant over time)

F_ITT _– failure rate estimated by intention-to-treat approach

GLURP *P. falciparum *glutamate rich protein

g_i _– proportion of the total recurrences which are true recrudescences at time t_i_

ind_i _– number of indeterminate PCR results at time t_i_

ITT – Intention-to-treat analysis

KM – Kaplan-Meier survival analysis

MSP1 *P. falciparum *merozoite surface protein 1

MSP2 *P. falciparum *merozoite surface protein 2

N – total number of patients

N' – adjusted total number of patients (effective sample size)

n_i _number of patients observed at time t_i_

or_i _– number of observed recurrences at time t_i_

PCR polymerase chain reaction

p – proportion of subjects beyond any follow-up time t who have not developed a recrudescence

PP – "per protocol" analysis

R – cumulative probability of developing a patent recrudescence

r_i _– patients with recrudescent infections at time t_i_

t_1/2 _– antimalarial drug elimination half life

## Authors' contributions

This was written jointly by the two authors

## Appendix 1

### Example: Analysing a non-inferiority trial

In a study, patients were randomly assigned treatment A or B and were followed up for 63 days. The aim of the study was to show that the new treatment (A) is not less effective than the standard treatment (B). *A priori *a margin of clinical non-inferiority (δ) had to be selected, which is defined as the largest reduction in efficacy which would be clinically acceptable.

As we are interested in non-inferiority of treatment A, the 95% CI for the difference in efficacy (A-B) should be less than the value – δ.

In this example a δ of 0.1 (i.e. 10%) was selected. There were 100 patients in each group. For those who were lost to follow-up or had a recurrence of infection we list the length of follow-up completed in the table below. Stars denote loss to follow-up. In treatment group A six failures were observed and six patients were lost to follow-up, while in group B four failures were observed and three patient were lost to follow-up.

A; 14* 14* 22  22 * 28* 28* 28* 34 44 52 61 63

B; 14* 14* 17  24   28*  29   43

Using the Kaplan-Meier method we estimate the efficacy as 0.94 (0.86 to 0.97) for treatment A and 0.96 (0.89 to 0.98) for treatment B.

The difference in efficacy (absolute risk reduction) A-B is -0.02. To calculate the confidence interval around this difference we need to calculate the 'effective' sample sizes: n_A _= 88/0.94 = 94 and n_B _= 93/0.96 = 97. Using Newcombe's formula we obtain a 95% CI for the difference of (-0.09 to 0.05).

**As the confidence interval is more positive than the δ of – 0.1 we conclude that treatment A is not inferior to treatment B. **But it should be noted that even with 100 patients per group the trial is underpowered; the precision of the cure rate estimates is poor (both confidence intervals for the individual group cure rates cross the 90% boundary) – see Figure 5.
